# Choroidal thickness and peripapillary RNFL in relation to axial length and progression in myopic children

**DOI:** 10.3389/fbioe.2026.1777062

**Published:** 2026-04-22

**Authors:** Zixun Wang, Xiaoxue Hu, Xiaoling Zhang, Yuhang Wang, Zhiqing Li, Zheng Guo

**Affiliations:** 1 Tianjin Key Laboratory of Retinal Functions and Diseases, Tianjin Branch of National Clinical Research Center for Ocular Disease, Eye Institute and School of Optometry, Tianjin Medical University Eye Hospital, Tianjin, China; 2 Wuhan Children’s Hospital (Wuhan Maternal and Child Healthcare Hospital), Tongji Medical College, Huazhong University of Science & Technology, Wuhan, Hubei, China; 3 Handan Eye Hospital (The Third Hospital of Handan), Handan, Hebei, China

**Keywords:** axial length progression, choroidal thickness, myopia, optical coherence tomography, prediction model, retinal nerve fiber layer

## Abstract

**Background:**

Axial length (AL) elongation is a key structural hallmark of myopia progression in children. Identifying early ocular structural characteristics associated with AL growth may help improve risk stratification, although robust predictive models remain limited.

**Purpose:**

To explore cross-sectional associations between baseline ocular structural parameters and AL, and to preliminarily evaluate the predictive potential of these parameters for rapid AL progression in myopic children.

**Methods:**

A total of 463 myopic children were retrospectively followed for 1 year. Baseline assessments included cycloplegic refraction, ocular biometry, and optical coherence tomography (OCT)–derived measurements of choroidal thickness (ChT) and retinal nerve fiber layer (RNFL). Rapid AL progression was defined as an AL increase of >0.2 mm over 1 year. Cross-sectional correlations between baseline AL, ChT, and RNFL parameters were analyzed. Least absolute shrinkage and selection operator (Lasso) regression with 10-fold cross-validation was used for feature selection, followed by the development and validation of a logistic regression model. Model performance was assessed using the area under the receiver operating characteristic curve (AUC), calibration analysis, and decision curve analysis (DCA).

**Results:**

At baseline, AL showed significant cross-sectional associations with ChT and multiple RNFL parameters, with the strongest correlations observed for ChT and nasal–inferior RNFL thickness (RNFL[NI]). Lasso regression retained age, baseline AL, ChT, and RNFL[NI] as candidate predictors of rapid AL progression. In the validation set, the final model demonstrated modest discriminative performance (AUC = 0.703, 95% CI: 0.598–0.801) with acceptable calibration. Decision curve analysis indicated limited but consistent net clinical benefit across a range of threshold probabilities.

**Conclusion:**

This study provides exploratory evidence that posterior segment structural parameters are associated with AL and may be useful for predicting subsequent AL progression in myopic children. Although the model’s predictive performance was modest, the findings support the hypothesis that OCT-derived biomarkers could contribute to future risk-stratification strategies, warranting further validation and refinement.

## Introduction

Myopia among children has become a major public health issue of global concern (Naidoo et al., 2019; Yang et al., 2025; Chen Z. et al., 2023). A recent national study indicates that by 2025, China’s overall myopia rate and high myopia (HM) rate are expected to reach 61.3% and 17.6%, respectively (Pan et al., 2025). As myopia progresses to HM, it may cause irreversible damage to the retina and optic nerve, a significant cause of blindness (Yii et al., 2024; Burton et al., 2021). Therefore, researchers are increasingly shifting myopia management to earlier stages to identify key factors influencing myopia progression and intervene promptly, thereby reducing irreversible vision impairment caused by myopia (Grzybowski et al., 2020; Cai et al., 2025; Han et al., 2022). However, there is currently a lack of data on changes in the fundus and optic nerves in children with early myopia as their myopia and axial length (AL) progress.

Changes in AL inevitably lead to alterations in the fundus and optic nerves from a biomechanical perspective (Liu et al., 2025; Tao et al., 2025). Previous studies have demonstrated that choroidal parameters can predict progression and pathological changes associated with high myopia, indicating their influence on myopia progression and AL elongation (Zeng et al., 2024; Hiraoka et al., 2025). Choroidal thickness (ChT) is currently recognized as one of the key indicators in myopia control (Wang et al., 2024; Hu et al., 2026). However, in the visual pathway, retinal nerve fiber layer (RNFL) thickness is a critical parameter. Previous research has shown that structural alterations in the RNFL are associated with glaucoma-related damage (Saito et al., 2024; Kim et al., 2024). Although these studies were conducted in glaucomatous populations, they highlight the structural vulnerability of the RNFL, which may also be relevant to AL elongation in myopia (Li et al., 2024; Jeoung et al., 2020; Dai et al., 2025). To date, evidence regarding the correlation between AL, ChT, and RNFL in children with myopia remains insufficient.

Current studies on RNFL changes and optic neuropathy associated with the long-term progression of childhood AL have demonstrated a close association between RNFL and AL (Eppenberger et al., 2026; Jonas et al., 2025; Yamashita et al., 2025). These studies focus on lesions as outcomes (such as the onset of glaucoma or pathological changes in the optic nerve). Whether baseline RNFL information in myopic children can provide clinically meaningful insights into the future progression of individual AL remains unclear. The potential predictive value of early choroidal parameters and RNFL in myopic children for dynamic changes in AL has not been explored.

Therefore, the objective of this study is to investigate differences and correlations between ChT and RNFL in children with uninterrupted myopia progression under natural conditions, specifically examining variations in AL and myopic states. We also employed a longitudinal cohort to construct a predictive model for axial length progression using meaningful baseline data, thereby substantiating its potential diagnostic value.

## Methods

### Data collection and ethics statement

The study was approved by the Ethics Committee of Wuhan Children’s Hospital and conducted in accordance with the principles outlined in the Declaration of Helsinki (20211126-E04, 20211126-E05, 2024R109-F01). All children included in the study were followed for 1 year. This was a retrospective cohort study conducted at Wuhan Children’s Hospital and Tianjin Medical University Eye Hospital between January 2023 and July 2025. Among the 463 children included, only the right eye of each participant was analyzed to avoid interocular correlation bias. All methods were performed in accordance with relevant guidelines and regulations. Informed consent was obtained from the children and their guardians for all procedures involving comprehensive physical examinations and ophthalmic assessments. Children with systemic diseases (e.g., congenital heart disease, ocular trauma, or ophthalmic diseases such as glaucoma, cataracts, infectious eye infections, and strabismus) were also excluded. Additionally, we inquired in detail about whether participants had undergone myopia control treatments. We also excluded them (e.g., orthokeratology treatment, red light therapy, low-concentration atropine, defocus lens therapy).

### Baseline eye examination

All baseline refractive data were obtained under cycloplegia. The specific operational process is the same as previously reported by our team (Hu et al., 2025). For baseline information on children, we included the following indicators: (1) Patient basic information: sex, age; (2) Refractive information: Cycloplegic Sphere Equivalent (SE), Cycloplegic Diopter Sphere (DS), Cycloplegic Diopter Cylindrical (DC), AL, Keratometry (K) (including K1 and K2), Anterior chamber depth (ACD); (3) Optical Coherence Tomography (OCT) examination, including ChT measurement and RNFL assessment. The ChT was defined as the vertical distance between the hyperreflective lines of the retinal pigment epithelium and the choroid/sclera border at the central fovea. The ChT was measured twice by two ophthalmologists using the OCT system software. Inter-observer agreement was evaluated prior to analysis, and discrepancies greater than 10 μm were re-measured to ensure measurement reliability. The mean of the two measurements of each patient and control subject was used for the analysis. RNFL measures the thickness at six positions (Temporal Superior (TS), T Inferior (TI), Nasal S (NS), NI, N, and T) centered on the optic nerve. Refraction was performed three times using an autorefractor (KR-800, Topcon, Tokyo, Japan). AL, K1, and K2 were measured using partial coherence interferometry IOL Master (Carl Zeiss 500, Meditec, Oberkochen, Germany). ChT and RNFL were obtained using Spectralis OCT (Heidelberg Engineering, Heidelberg, Germany).

### Model development and validation

We defined 1 year AL progression ≤0.2 mm as the AL nonprogressive group and AL progression >0.2 mm as the myopic AL progressive group. This cutoff was selected as a pragmatic threshold based on prior pediatric myopia studies and on the use of a value around 0.2 mm/year to represent clinically meaningful or excessive AL elongation in school-aged children. We also acknowledge that the interpretation of a fixed threshold may vary with age; therefore, this definition should be regarded as a practical rather than an age-standardized classification (Hu et al., 2025; Graff et al., 2024; Du et al., 2025; Lanca et al., 2021). The sample subjects were divided into a training set and a validation set (training set: validation set = 8:2). Due to the large number of variables after image quantization, the lasso was used to select features, which is a method to introduce L1 regularization, select features, and reduce dimensions by compressing coefficients, screening features with significant contributions, and eliminating redundant features.

This study included baseline information such as sex, age, cycloplegic SE, DS, DC, Flat K (K1), and Steep K (K2), AL, ChT, and RNFL in N, NI, NS, T, TS, and TI. The Lasso regression model selects features with nonzero coefficients as potential predictors, effectively reducing multicollinearity and preventing overfitting (Li et al., 2023). We employed Lasso regression with 10-fold cross-validation to analyze the initial high-dimensional dataset and identify relevant variables.

Model training is conducted on the designated training set, while model validation is performed on a separate, independent validation set. The two processes are mutually independent. Logistic regression (LR) was used to develop predictive models. The configuration yielding the highest area under the receiver operating characteristic curve (AUC) was selected as the optimal model. Model performance was quantified using the AUC, sensitivity, specificity, and F1 score (Nie et al., 2026). To further assess the clinical applicability of the models, a decision curve analysis (DCA) was performed (Chalkou et al., 2023). DCA evaluates the net clinical benefit of a predictive model across a range of threshold probabilities, thereby demonstrating its value in guiding clinical decisions (Chen et al., 2026).

A clinical prediction model was developed to estimate the probability of rapid AL progression in myopic children. Selected variables were incorporated into a logistic regression model and fitted using the training dataset. A risk chart was constructed using a nomogram to visually represent the contributions of variables and provide personalized risk assessments for inadequate axial length control. Predicted probabilities were derived from linear predictions via the plogis function and calibrated across a 0.001–0.999 multi-threshold range in the validation set. The interactive dynamic nomogram was implemented using the regplot and DynNom packages. The Hosmer-Lemeshow goodness-of-fit test (HL test) was used to assess the fit between the predicted values from the logistic regression model and the observed outcomes. The test was performed independently on both the training and validation sets to verify the model’s stability and generalization (Qu et al., 2026).

### Statistical analysis

Continuous variables were tested for normality using the Shapiro-Wilk (S-W) test. Data meeting normal distribution were presented as mean ± SD, while non-normal distributions were shown as median [P25, P75]. Age was treated as a continuous variable and presented as the median [P25, P75] due to non-normality. Categorical variables were presented as counts and percentages. All statistical analyses were performed using GraphPad Prism 9.0 software (GraphPad Software, La Jolla, CA, United States). We conducted a simple linear correlation analysis to examine the relationships among AL, ChT, and nerve fiber parameters. Group comparisons were performed using Student’s t-test for normally distributed variables or the Wilcoxon rank-sum test for non-normally distributed data. Categorical variables were presented as counts and percentages, and group differences were evaluated using the chi-square or Fisher’s exact test, as appropriate. *p* < 0.05 was considered significant.

## Results

### Baseline characteristics

As shown in Table 1, a total of 463 subjects were included in this study and were divided into two groups (ΔAL ≤0.2 mm, n = 238; ΔAL >0.2 mm, n = 225). No significant difference in sex distribution was observed between the two groups (p = 0.942). However, significant intergroup differences (*p* < 0.05) were found in age, AL, corneal curvature (K1 and K2), ChT, and the nerve fiber layer parameter NI. Specifically, age, AL, and ChT showed highly significant differences (*p* < 0.01).

**TABLE 1:** Baseline information included in this study.

Characteristic	Level	Overall	ΔAL ≤0.2 mm	ΔAL >0.2 mm	p
n	​	463	238	225	​
Sex (%)	Male	311 (67.17)	159 (66.81)	152 (67.56)	0.942
​	Female	152 (32.83)	79 (33.19)	73 (32.44)	​
Age (y)	​	8.00 [7.00, 9.00]	8.00 [7.00, 10.00]	8.00 [7.00, 9.00]	**<0.01**
AL (mm)	​	24.48 [23.90, 25.17]	24.69 [24.14, 25.30]	24.29 [23.55, 24.93]	**<0.01**
K1 (D)	​	42.61 (1.52)	42.43 (1.52)	42.80 (1.50)	**0.01**
K2 (D)	​	44.05 (1.68)	43.87 (1.68)	44.23 (1.67)	**0.022**
SE (D)	​	−2.25 [-3.69, −1.25]	−2.38 [-3.72, −1.50]	−2.00 [-3.63, −1.00]	0.104
DS (D)	​	−2.00 [-3.25, −1.00]	−2.00 [-3.25, −1.25]	−1.75 [-3.25, −0.75]	0.127
DC (D)	​	−0.50 [-1.00, −0.25]	−0.50 [-1.00, −0.50]	−0.50 [-1.00, 0.00]	0.419
IOP (mmHg)	​	14.80 [13.00, 16.80]	14.55 [12.93, 16.80]	15.00 [13.00, 17.00]	0.451
ChT (um)	​	255.00 [221.00, 297.00]	241.00 [210.12, 277.62]	271.00 [236.50, 311.00]	**<0.01**
TS (um)	​	149.00 [137.00, 162.00]	150.00 [138.00, 163.00]	149.00 [135.00, 161.00]	0.369
T (um)	​	87.00 [77.00, 96.00]	87.00 [78.00, 96.75]	86.00 [77.00, 95.00]	0.189
TI (um)	​	155.00 [144.00, 166.00]	155.00 [146.00, 167.00]	155.00 [143.00, 166.00]	0.582
NS (um)	​	116.00 [103.00, 129.00]	116.00 [101.25, 129.00]	117.00 [103.00, 129.00]	0.687
N (um)	​	59.00 [51.00, 67.00]	59.00 [51.00, 66.75]	59.00 [52.00, 67.00]	0.876
NI (um)	​	102.00 [92.00, 115.00]	102.00 [94.25, 117.75]	101.00 [91.00, 114.00]	**0.044**

Values are presented as mean ± SD, for normally distributed variables and median [P25, P75] for non-normally distributed variables. P values were calculated using Student’s t-test, Wilcoxon rank-sum test, or chi-square test as appropriate.

### Correlation between baseline AL and other parameters

**FIGURE 1:**
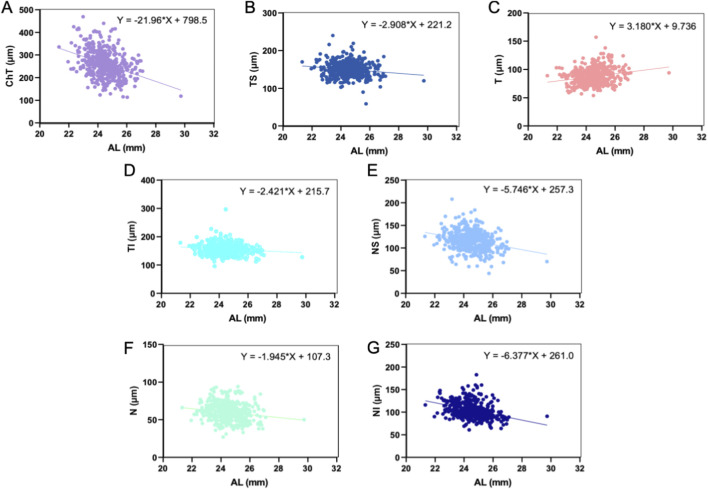
Linear relationship and equations between ChT **(A)**, TS **(B)**, T **(C)**, TI **(D)**, NS **(E)**, N **(F)**, NI **(G)**, and AL.

**FIGURE 2:**
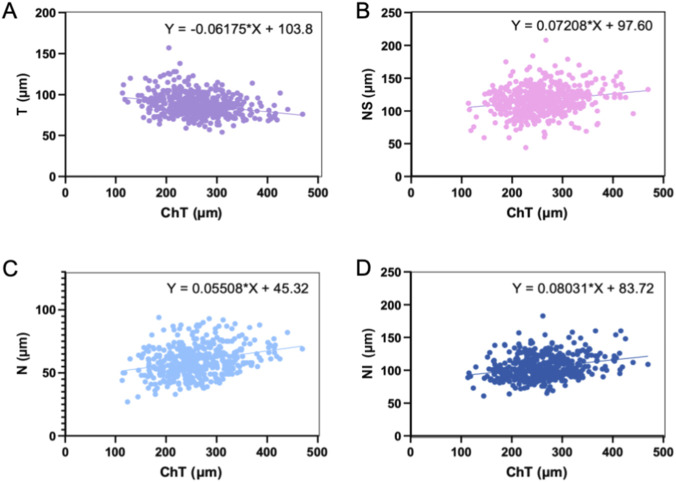
Linear relationship and equations between T **(A)**, NS **(B)**, N **(C)**, NI **(D)**, and ChT.

The univariate linear regression analysis in Table 2 demonstrates significant correlations between AL and all parameters (ChT, TS, T, TI, NS, N, NI) (*p* < 0.01), with ChT showing the strongest correlation (*r* ^2^ = 0.1367), indicating a statistically significant but modest explanatory effect. Among the parameters related to nerve fiber layer thickness, AL showed the strongest correlation with NI (*r* ^2^ = 0.1286). ChT exhibits significant relationships with T, NS, N, and NI (*p* < 0.01), but no significant correlations with TS or TI. Figures 1, 2 illustrate the linear relationships and regression equations between AL and ChT, TS, T, TI, NS, N, and NI, and between ChT and T, NS, N, and NI. Among the RNFL sectors, RNFL[NI] showed the strongest association with baseline AL and was therefore considered the most informative sector-specific RNFL candidate for subsequent model development.

**TABLE 2:** Single-linear Regression Analysis of AL, ChT, and nerve fiber parameters.

Variable	*r* ^2^	P (single-linear)
AL		
ChT	0.1367	**<0.01**
TS	0.0221	**<0.01**
T	0.0517	**<0.01**
TI	0.0161	**<0.01**
NS	0.0728	**<0.01**
N	0.0268	**<0.01**
NI	0.1286	**<0.01**
ChT		
TS	0.0006	0.6042
T	0.0688	**<0.01**
TI	0.0005	0.6320
NS	0.0404	**<0.01**
N	0.0758	**<0.01**
NI	0.0720	**<0.01**

Bold values in the P value column indicate statistically significant associations (P < 0.05).

### Model validation and application

**FIGURE 3:**
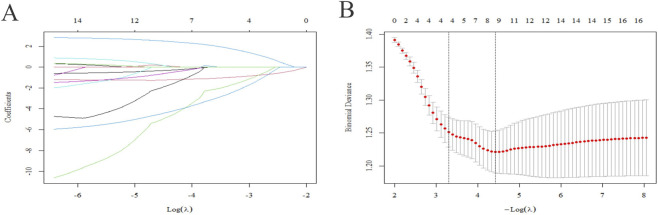
Lasso regression for variable selection in AL progression children. **(A)** Coefficient path plot: Each curve represents the trajectory of a candidate variable’s coefficient as the regularization parameter λ increases (plotted against log(λ)). Coefficients are progressively shrunk toward zero with increasing λ; the numbers at the top indicate the count of non-zero coefficients at each λ level. **(B)** Binomial deviance curve from 10-fold cross-validation: The red dots denote the mean deviance across folds for each λ (plotted against −Log(λ)), and vertical dashed lines indicate λmin (left) and λ1se (right; selected to prioritize model parsimony). Error bars represent the standard error of deviance across folds.

To identify a parsimonious set of predictors while mitigating overfitting, Lasso regression (coupled with 10-fold cross-validation) was employed for variable selection. Cross-validation yielded two key regularization parameters: a minimum λ of 0.01203884 and a 1-standard-error λ of 0.03676488 (Figure 3B). The coefficient path plot (Figure 3A) depicts the shrinkage trajectory of each candidate variable’s coefficient across varying log(λ) values. Consequently, four predictors (age, AL, ChT, RNFL[NI]) were retained for subsequent model development, balancing predictive performance and interpretability. This selection was also consistent with the baseline analyses, in which RNFL[NI] showed the strongest RNFL–AL association and the clearest between-group signal among the RNFL sectors.

**FIGURE 4:**
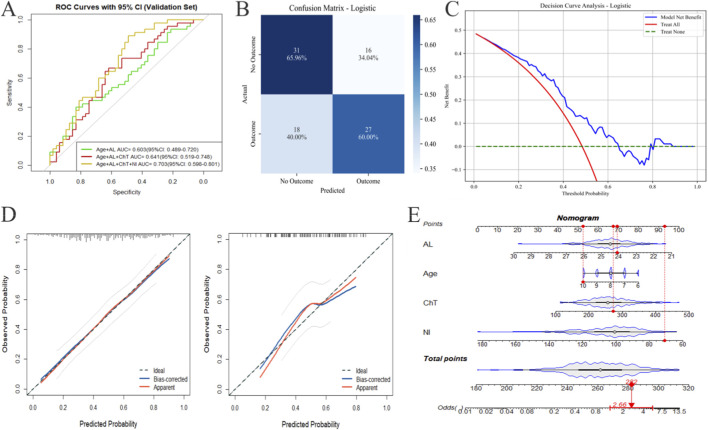
Predictive model performance for AL progression in children. **(A)** Validation-set receiver operating characteristic (ROC) curves for models with varying combinations of variables (e.g., Age + AL + ChT + RNFL [NI]) show corresponding areas under the curve (AUCs); the final model achieved an AUC of 0.703 (95% confidence interval: 0.598–0.801). **(B)** The confusion matrix of the final model reports counts (and percentages) of true negatives (31, 65.96%), false positives (16, 34.04%), false negatives (18, 40.00%), and true positives (27, 60.00%) in the validation set. **(C)** Decision curve analysis shows the model’s net benefit (blue curve) exceeds that of the “Treat None” strategy (red curve) across most threshold probabilities, indicating clinical utility. **(D)** Calibration plots compare predicted and observed outcome probabilities: the bias-corrected curve (adjusted via bootstrapping) aligns closely with the ideal 45° line, confirming good model calibration. **(E)** The nomogram translates variables (AL, Age, ChT, RNFL[NI]) to point values; total points map to predicted outcome odds (e.g., ∼260 total points correspond to an odds ratio of ∼2.69).

To investigate the impact of different types of variables on model accuracy, we built three models: Model one included only age and AL, Model 2 added ChT, and Model 3 finally included RNFL[NI]. In the validation set, the Model 3 (incorporating Age, AL, ChT, and NI) achieved an AUC of 0.703 (95% confidence interval [CI]: 0.598–0.801). For comparison, alternative models exhibited lower AUCs (0.696 and 0.641, respectively), confirming the goodness of the final model (Figure 4A). Corresponding performance metrics included an accuracy of 0.630, a precision of 0.628, a sensitivity of 0.600, a specificity of 0.660, and an F1 score of 0.614 (Figure 4B).

We selected the model with an AUC of 0.703 for subsequent performance evaluation, including confusion matrix analysis, DCA, nomogram construction, and stability validation. The DCA evaluated the net benefit of the final model across a range of threshold probabilities. The model’s net benefit curve (blue line) exceeded that of the “treat none” strategy (green line) across most threshold probabilities (0.0–0.8), indicating that applying the model to guide clinical decisions would yield greater benefit than either treating no patients or treating all patients (Figure 4C).

A nomogram (Figure 4E) was developed to visually translate the final model into a clinical tool: each variable (AL, Age, ChT, RNFL[NI]) was assigned a point value, and the total points were mapped to the predicted probability of the outcome. For example, a patient with median values of all variables would accumulate ∼260 total points, corresponding to an odds ratio of ∼2.69 for the outcome. This nomogram provides a user-friendly method for individualized risk prediction. Calibration plots (Figure 4D) assessed the agreement between predicted probabilities and observed outcomes in the training set (*X* ^2^ = 1.90, *p* = 0.98) and the validation set (*X* ^2^ = 4.74, *p* = 0.79). The “bias-corrected” curve (adjusted via bootstrapping) closely aligned with the ideal 45° line, indicating good calibration (predicted probabilities were well-calibrated to actual event rates). This consistency confirmed the model’s stability in the validation set.

## Discussion

In this study, we developed and validated a clinical prediction model to estimate the risk of rapid AL progression in myopic children over a 1-year period by integrating baseline ocular biometric and structural parameters. We found that age, baseline AL, ChT, and the RNFL[NI] were independently associated with subsequent AL progression and together provided the best predictive performance. Compared with models based solely on demographic and refractive parameters, the inclusion of ChT and RNFL[NI] significantly improved discrimination, underscoring the added value of posterior-segment structural biomarkers in assessing early myopia progression. The final model demonstrated modest discrimination, good calibration, and a limited but consistent net benefit on decision curve analysis, suggesting that baseline OCT-derived structural information may provide complementary value for individualized risk stratification of axial elongation in myopic children.

Importantly, the modest validation performance of our model should be interpreted in the context of the broader myopia-prediction literature. A recent systematic review showed that prediction models for myopia have incorporated refractive, biometric, environmental, genetic, and integrated predictors, yet few are sufficiently validated for routine clinical application. In parallel, ophthalmic prediction studies using more heterogeneous ocular metrics have reported higher discrimination. For example, Xiao et al. developed an integrative model for orthokeratology lens decentration that incorporates multiple corneal morphological parameters and age, and reported a logistic regression AUC of 0.82 (Xiao et al., 2024a; Wang et al., 2026a; Wang et al., 2026b). Although lens decentration is a different endpoint from axial elongation, that study illustrates the potential value of integrating diverse ocular metrics and comparing alternative model architectures. In contrast, our model was intentionally restricted to a small set of posterior-segment OCT parameters and baseline clinical variables, without corneal topography, OCTA, fundus imaging, or environmental exposures, which likely contributed to the more modest discrimination observed here.

The results of this study showed that ChT gradually decreased as AL increased, consistent with previous studies. Zhuo Y et al. found that the ChT of highly myopic macaques was smaller, and that the AL significantly contributed to the reduction in ChT (Ye et al., 2025). Chen K’s team examined 520 eyes from 263 cynomolgus monkeys, revealing that AL increased while ChT decreased as SE decreased (Chen K. et al., 2023). Hansen NC et al. demonstrated that longer eyes had significantly thinner ChT in all choroidal sectors (Hansen et al., 2024). XU X and his team discovered that choroidal thinning becomes more pronounced in the central and perimacular regions as the AL increases (Xie et al., 2022). Therefore, we hypothesize that the ChT measured below the fovea centralis in this study is more sensitive to AL changes. The correlation coefficient with AL was statistically significant (p < 0.01), with ChT showing the highest correlation (*r* ^2^ = 0.1367). Although the magnitude of correlation was moderate, this finding is consistent with the multifactorial nature of axial elongation, in which structural biomarkers are expected to explain only part of the variance. Our study also unexpectedly revealed correlations between ChT and RNFL parameters T, NS, N, and NI. Specifically, ChT showed a negative correlation with RNFL[T] and a positive correlation with other variables. This finding is consistent with the results of Zhang Z et al.‘s meta-analysis, which showed differences in temporal and other directional pRNFL measurements between the low-to-moderate myopia and high myopia groups (Zhang et al., 2023). The specific reasons will be analyzed in the discussion that follows.

The correlation analysis between baseline AL and ChT with the RNFL revealed that RNFL[T] showed a positive correlation with AL and a negative correlation with ChT. This finding was inconsistent with other RNFL directions. The remaining five RNFL directions exhibited negative correlations with AL and either positive correlations or no correlation with ChT. Takehiro et al. reported findings like ours. Our study builds upon theirs by conducting a more comprehensive investigation in terms of sample size and the correlation with ChT (Yamashita et al., 2025). Normal-tension glaucoma causes localized damage to nerve fibers, particularly in the temporal region of the optic disc, and myopia is a known risk factor. In China, myopia is highly prevalent, and the incidence of normal-tension glaucoma is very high (Kawase et al., 2008). Bak E et al. propose that as AL progresses, changes in the scleral screen structure lead to RNFL[T] crowding, potentially evolving into pre-glaucomatous changes. Additionally, studies have reported that RNFL[T] changes associated with AL elongation can cause optic disc torsion and peripapillary atrophy (Bak et al., 2020; Zhang et al., 2024). Cheng et al. also found that RNFL[T] is a retinal parameter in adult pathological myopia (Cheng et al., 2022). Compared to previous studies, our research examines changes in the RNFL during the early progression of AL in childhood myopia.

Furthermore, RNFL[T] correlates with both ChT and total AL. Our findings may, from two perspectives, confirm that ChT influences AL, and that AL subsequently affects biomechanical alterations in RNFL[T]. Notably, this occurs during the early stages of childhood myopia, underscoring the critical importance of advancing RNFL monitoring and updating our understanding of this relationship.

An interesting finding of this study was the consistent association between AL progression and thinning of the nasal–inferior RNFL sector. Rather than indicating primary neural injury, this sector-specific association may more plausibly reflect regional susceptibility to biomechanical remodeling during ocular elongation (Leung et al., 2006). In axial myopia, posterior pole expansion is not spatially uniform, and displacement of the Bruch’s membrane opening as well as optic nerve head remodeling may preferentially involve the inferior and nasal peripapillary regions. Because the RNFL[NI] lies at the intersection of these mechanically vulnerable areas, it may be particularly sensitive to early structural alterations accompanying axial elongation (Leung et al., 2006). Comparable observations have also been reported in school-aged and adolescent populations, supporting the possibility that sector-specific peripapillary RNFL remodeling begins early in myopic eye growth (Cui et al., 2021). In addition, inferior retinal regions may be more vulnerable to concomitant choroidal thinning and microvascular alteration during axial elongation, which could further contribute to localized RNFL remodeling. In our study, RNFL[NI] was not only associated with AL, but also showed a concurrent relationship with choroidal thickness, supporting the interpretation that NI thinning may represent a secondary structural response to axial growth rather than direct neurodegeneration (Eppenberger et al., 2026). Consistent with this interpretation, RNFL[NI] showed the strongest RNFL–AL association at baseline, was the only RNFL sector to exhibit a significant between-group difference, and was retained in the final model after Lasso selection. Taken together, these findings suggest that RNFL[NI] may serve as a relatively sensitive structural correlate of early axial elongation and provide incremental information for future AL prediction models.

To investigate parameters across different dimensions (baseline information, choroidal parameters, optic nerve parameters), this study constructed traditional logistic regression models using distinct sets of variables. It calculated the AUC for independent validation sets. This enabled visualization of the importance of baseline variables for AL progression. To assess the incremental value of posterior-segment structural biomarkers, we compared nested logistic regression models that sequentially incorporated baseline clinical variables, ChT, and RNFL[NI]. Adding ChT and RNFL[NI] resulted in a modest improvement in discrimination in the validation set, suggesting that these OCT-derived parameters may provide complementary information beyond age and baseline AL alone. However, given the moderate overall model performance, these findings should be interpreted as exploratory and hypothesis-generating rather than sufficient for immediate clinical implementation.

This study has certain limitations. First, although incorporating RNFL[NI] and ChT improved model performance compared with simpler models, the overall discriminative ability remained modest. Therefore, the present findings should be interpreted as preliminary evidence supporting the potential relevance of OCT-derived structural biomarkers, rather than as definitive proof of clinical predictive utility. In the future, we plan to include more fundus-related parameters to construct a more robust model for predicting AL progression in children (Zhang et al., 2025). Second, this retrospective cohort study had a limited observation period of only 1 year. Future prospective studies should be conducted based on the key biological parameters identified, with continuous follow-up (Wang et al., 2026c). Additionally, the absence of multimodal parameters, such as OCTA and fundus imaging, among the included variables contributed to the suboptimal overall predictive performance. We plan to initiate large-scale, multimodal studies on the development and progression of AL in children. Additionally, important non-ocular determinants of myopia progression were not collected in this study. These include not only hereditary and behavioral factors, such as parental myopia, near-work intensity, and time spent outdoors, but also potential nutritional and systemic influences. Myopia progression is a multifactorial process, and systemic exposures may contribute to ocular growth through metabolic, inflammatory, and oxidative pathways. For example, an NHANES-based study of American adolescents reported that only cis-β-carotene showed a significant association with myopia, whereas most other measured micronutrients were not significantly associated, underscoring the complexity of these relationships rather than a simple nutrient-specific effect (Xiao et al., 2024b). Future prediction studies should therefore incorporate a broader set of non-ocular variables, including hereditary, behavioral, dietary, and systemic factors, to better reflect the multidimensional nature of AL elongation.

## Conclusion

This study demonstrates that baseline age, AL, ChT, and RNFL[NI] can be integrated into an interpretable model to estimate the risk of rapid AL progression in myopic children. Although the predictive performance remained modest, the inclusion of OCT-derived structural biomarkers provided incremental information beyond baseline clinical variables and may inform future model refinement. Further validation in larger and prospective cohorts is warranted before clinical implementation.

## Data Availability

The raw data supporting the conclusions of this article will be made available by the authors, without undue reservation.
